# Organizational design for strengthening community-based tourism: Empowering stakeholders for self-organization and networking

**DOI:** 10.1371/journal.pone.0294849

**Published:** 2024-01-23

**Authors:** Natalia Romero–Medina, Emily Flores–Tipán, Mauricio Carvache-Franco, Orly Carvache-Franco, Wilmer Carvache-Franco, Ruth González-Núñez

**Affiliations:** 1 Facultad de Ciencias Sociales y Humanísticas, Escuela Superior Politécnica del Litoral, ESPOL, Guayaquil, Ecuador; 2 Universidad Espíritu Santo, Samborondón, Ecuador; 3 Facultad de Economía y Empresa, Universidad Católica de Santiago de Guayaquil, Guayaquil, Ecuador; 4 Facultad de Ciencias Sociales, Educación comercial y Derecho, Universidad Estatal de Milagro, Milagro, Ecuador; University of Naples Federico II: Universita degli Studi di Napoli Federico II, ITALY

## Abstract

The San Pablo community in El Triunfo, Ecuador, is emerging as a promising community-based tourism destination. Despite its potential, the lack of knowledge about self-organization and its implementation has hindered effective tourism management. To address this challenge, a participatory approach was employed, involving the community and key stakeholders, such as the local government of El Triunfo. Through the utilization of Design Thinking and both online and in-person interviews, it was identified that an organizational structure based on networks and a culture of self-organization can drive local tourism. These aspects were incorporated into a Design Thinking-guided process, contributing to the understanding of how to forge an appropriate organizational framework for the community. Furthermore, this study aims for broader impact. The goal is not only to strengthen tourism in San Pablo, but also to inform the management of strategies and policies in other entities. The findings offer valuable insights to similar communities in Ecuador and the region. Collectively, this research enhances the comprehension of community-based tourism and proposes practical solutions for optimizing its management in emerging contexts.

## 1. Introduction

In the 1980s and 1990s, a new form of tourism emerged in Latin America: community-based tourism, which arose as an alternative for sustainable tourism development [1]. Since then, this type of tourism has expanded to other parts of the world, becoming an increasingly common practice in countries across different continents. Despite its growth, there remains a lack of research that specifically addresses theories of networks or self-organization within the context of community-based tourism. Therefore, this article aims to fill this gap in the literature and present new findings that contribute to the strengthening of community-based tourism in Ecuador.

According to the UNWTO [2], community-based tourism involves the engagement of local communities in the management and operation of tourism projects, providing them with the opportunity to gain economic benefits while simultaneously preserving their cultural traditions and the environment. In addition to these advantages, Spenceley and Meyer [3] posit that tourism serves as a development strategy aimed at enhancing living conditions through job creation, strengthening community organizations, and safeguarding cultural and natural heritage.

Despite efforts and achievements in the realm of community-based tourism, significant challenges in its sustainable development persist. In Ecuador, community-based tourism stands as a significant tool that allows local communities to improve their living conditions while simultaneously safeguarding and valuing their cultural and natural resources due to the country’s rich cultural and natural diversity. However, according to statistics from 2017 by the Pluractional Federation of Community Tourism of Ecuador (FEPTEC), only 42.85% of community-based tourism enterprises remain active, while the rest opt not to continue their operations due to two main factors: the emigration of community leaders and a lack of understanding about self-management in tourism [4].

This lack of understanding about what self-management entails and how to implement it is a cause for concern for communities that choose to engage in this type of tourism. In addition to this, Ruiz et al. [5] point out that not all communities possess the same level of organization and collective capacity in this field, as some lack the necessary organizational, political, and cohesive structure to efficiently manage their tourism activities.

Given this reality, the significance of self-management as a tool to enhance community organization and improve tourism within the community stands out. Self-management empowers communities to take control of their tourism activities using the resources available to them, thereby increasing their resilience to external changes or economic crises [6] and enabling them to make decisions that benefit their social and economic development. In combination, self-management and community organization are pivotal for sustainable and enduring success in community-based tourism.

Thus, an organizational design based on two fundamental pillars is proposed: self-organization and networked collaboration. A crucial aspect of this design is that a well-organized community has greater opportunities to engage with other tourism stakeholders, potentially leading to new collaborations and alliances. Self-organization provides the necessary framework for establishing successful networking relationships, facilitating joint decision-making, and coordinated efforts among various actors, even those not directly part of the community.

This article is grounded in the significance of Organizational Design. In the context of community-based tourism, this topic becomes even more relevant, as the absence of a clear organizational structure can adversely affect coordination among key stakeholders and decision-making processes. Therefore, it is essential to address this gap and explore how the implementation of an Organizational Design under a participatory and synergistic management structure can benefit community-based tourism management in Ecuador.

In particular, a detailed analysis is conducted on the case of El Triunfo, an emerging city in community-based tourism in Ecuador, with a special focus on the San Pablo community. Within this community, stakeholders involved in community-based tourism have faced significant challenges in collaborating and organizing efficiently, which has hindered the achievement of sustainable success. Among the identified issues are unequal participation among key tourism actors, a lack of robust planning, and the absence of adequate support.

To address this challenge, the objectives of this article are I) Analyze the organizational situation of the San Pablo community to identify the present organizational and communicational challenges and needs within the tourism sector, and II) Determine an organizational model that promotes self-organization, integration, and coordination among stakeholders engaged in the community’s tourism activities.

Achieving these objectives aims to enrich the theoretical landscape and provide practical recommendations and strategies for enhancing collaboration and organization among local communities, the public sector, the private sector, and other key stakeholders. Furthermore, governmental authorities can utilize this information to formulate supportive policies and programs that foster sustainable community-based tourism, empowering communities and facilitating the creation of cooperation networks.

In combination, this study will provide valuable insights into how to address more effective, sustainable, and inclusive management of community-based tourism, both in Ecuador and in other regions facing similar challenges.

In pursuit of these goals, the article adheres to a well-defined structure. It initiates with an Introduction that establishes the context and relevance of the study. Proceeding further, the examination encompasses three pivotal theoretical concepts as part of the Literature Review. Subsequently, within the Methodology section, the study’s approach and the guiding principles of the Design Thinking process are meticulously expounded upon. The ensuing section presents the outcomes derived from this process. Ultimately, the conclusion encapsulates the implications of the findings and their role in fortifying community-based tourism, both within Ecuador and in a broader global context.

## 2. Literature review

### 2.1. Partnerships and networking in tourism

The development of a tourism area requires the formation of partnerships among local actors, as they can help to elevate local informal collaboration to the next level, create a new entity for tourism, and strengthen connections among local stakeholders [7]. A crucial factor in building an alliance is the creation of networks through associations, which requires good relationships among members and a high level of trust between them [8]. Particularly in Slovakia, Gajdošík et al. [9], conducted a study on two internationally important mountain destinations, which focuses on the implementation of innovations and networks in the tourism sector, through the analysis of a destination management system database and the interactions and intensity of relationships among stakeholders, resulting in the finding that formal and informal networks and collaboration among stakeholders contribute to the optimal development of the tourism destination in Slovakia. From this perspective, Kimbu & Ngoasong [10] conducted a case study in Cameroon to illustrate how power networks can influence the implementation of decentralization policies in tourism development, highlighting the importance of strengthening the capacity of local governments and community organizations in decision-making, through the promotion of collaboration and dialogue among key actors in the tourism industry, as well as investment in training and education programs. At the national level, Reyes et al. [11] proposed a model for managing community-based tourism by integrating the organization of cooperatives under work networks and building relationships with public-private actors to improve communication and relationships between indigenous communities and other external factors that influence the development of community-based tourism in the Amazonian province.

### 2.2. Organizational transformation for sustainable development

Although the concept of organizations is based on the voluntary union of individuals directing their efforts towards shared objectives, their success relies on synergy and a positive response to environmental stimuli. In this sense, organizations are considered social cells [12]. However, an inadequate structure can jeopardize the institution’s position and sustainability, diverting actions from their intended direction. It is within this dynamic that aspects such as organizational culture, work regulations, and organizational designs become relevant.

When the implemented organizational design doesn’t operate effectively, a transformation is needed. According to Hellriegel, Jackson, and Slocum [13], organizational change is linked to modifying the design or functioning of an organization [14]. These transformations can be driven by internal needs or the constant changes in the society in which we operate.

Implementing changes is challenging due to resistance, but it’s essential to correct inefficiencies. Strategic planning is crucial to guide this process. Appointing change agents is important as they foster trust and enhance communication. A successful example is Cotratudossa company in Cuenca, which improved its organizational culture and structure in the human resources department. A strategic plan, functional organizational chart, and procedures manual led to increased competitiveness.

### 2.3. Self-organization in destination management with a focus on community-based tourism

Self-organization in tourism refers to a process of tourism management based on the active and autonomous participation of the local community in decision-making and control of the destination [15]. In addition, according to Pavlovich K. [16] in his article "A rhizomic approach to tourism destination evolution and transformation”, tourist destinations are not simple hierarchical systems with a center of power and control, but rather decentralized and constantly changing systems. Using examples of tourist destinations in New Zealand and Australia, the author shows how tourist destinations can evolve and transform through collaboration and interconnection among diverse actors and resources. In this way, self-organization becomes key for proper management of a tourist destination, as it is a more collaborative and flexible approach according to the perspective of the mentioned article, and as presented by [17] in their project "Touristic Development and Relational Dynamics. Analysis Methodology for the Active Management of Tourist Destinations," where they mention that establishing relational dynamics is fundamental in the active management of tourist destinations, especially in emerging ones. They emphasize that good dynamics among the involved actors are vital for achieving set goals and objectives, allowing for quicker decision-making and active participation from all stakeholders. An example of this is the city of Úbeda, where there is no central leadership position; instead, responsibilities are shared, and autonomy or self-organization is encouraged in carrying out activities and tasks. This approach has enabled Úbeda to achieve significant tourism development compared to other destinations.

## 3. Methodology

### 3.1. Study area

The city of El Triunfo, located in the province of Guayas in Ecuador, is important as a connection point for various regions of the country due to its strategic location at the intersection of roads that connect to Guayaquil, Cuenca, and the central highlands. The region has rich vegetation, including tropical forests, mangrove forests, wetlands, grasslands, and shrublands, and is known for agricultural production with plantations of sugarcane, bananas, and rice for import and export.

In addition, the local community has developed tourism activities for visitors, including community-based tourism and ecotourism. Tourists can enjoy visits to communities, hikes along trails, bike rides, and the opportunity to try delicious local cuisine made from green plantains, such as fish bollo, corviche, cazuelas, and the traditional chanfaina made from pork viscera. Due to its privileged agricultural location, this activity has been linked to tourism, forming skilled artisans who work with natural products such as banana stems to make crafts and clothing from this material. Furthermore, El Triunfo is part of the Adventure Route, which combines adventure activities and experiences with the exploration of nature, cultural heritage, and other tourist attractions that can range from tourist haciendas, resorts, and even small community beaches.

In this regard, this work focuses on the community of San Pablo, located 15 km from the center of El Triunfo city, on the way to Huigra. In this community, where about 200 people live, the COVID-19 sanitary emergency provided them with an opportunity to resume their tourism activities because, being surrounded by nature, it became an ideal place for people who wanted to be away from the crisis caused by the pandemic. The activities include swimming and refreshing in a natural pool called "La Jungla" providing a visually stunning and relaxing environment, as well as hiking through the forest and opting for a natural experience in caves.

### 3.2. Methodology, data collection and analysis

The study had ethical approval from the Ethics Committee of the ESPOL Polytechnic University of Ecuador with Code: FCSH-14-2021. Informed consent was requested from all participants in writing at the beginning of the questionnaire. This study was carried out over a period of four months, from October 2022 to April 2023, to address the need for proper and specific tourism planning in the community of San Pablo, Ecuador. This community has faced issues related to a lack of organizational knowledge and other challenges in community-based tourism entrepreneurship, such as a lack of collaboration networks and strategic partnerships. The central objective of this research was to identify key constructs related to organizational design and its impact on strengthening community tourism in San Pablo, including the significance of networking for proper self-management and sustainable development in the tourism sector.

To achieve the stated objectives, the Design Thinking methodology was adopted, an iterative process comprising four stages: 1) Discover and empathize, 2) Analyze and define criteria, 3) Ideate and prototype, and 4) Experiment and validate. The process centers on cultivating empathy with project stakeholders and deeply understanding user needs to devise innovative solutions [18].

The four stages of the Design Thinking process were implemented through an exploratory research approach, enabling an understanding of the working environment, identification of issues and their causes, and proposal of solutions. For data collection, a qualitative research approach was employed using structured personal questionnaires. These questionnaires were designed based on a review of academic literature and were validated by a panel of three experts in tourism planning and organization. The interviews were conducted with representative members from the San Pablo community and the local government of El Triunfo. The participants were carefully selected through a small yet purposeful sampling method to ensure rich and meaningful information. Additionally, Design Thinking tools were applied to address and creatively resolve complex community problems while maintaining a user-centered focus.

Additionally, an organizational assessment method was employed, using appropriate scales to measure data and minimize errors in data collection. To complement the research with descriptive quantitative data, techniques such as direct and participant observation of the tourism offerings were used, along with comparative analysis with successful practices in the tourism sector within communities (benchmarking).

Below, each phase of the process is presented in detail, highlighting how it was applied in our study.

#### 3.2.1. First phase: Discovery and empathy

The first two stages of the Design Thinking approach focus on achieving the project’s first objective: discovering user problems and aspirations related to San Pablo and establishing effective empathy with them. To achieve this empathy, three in-depth semi-structured interviews were conducted with key representatives from the community. Two of these interviews were conducted online, involving the official from the Tourism Department of GAD El Triunfo and the leader responsible for tourism management in the San Pablo community. The third interview was conducted through a field visit, allowing for a closer interaction with the representative of the community’s main tourist attraction. This combination of online meetings and field visits facilitated a holistic and detailed perspective of the issues and needs related to community-based tourism in San Pablo.

To guide the interviews and gather relevant information, thirteen guiding questions were established (Table 1), addressing topics such as the primary objectives of community-based tourism for the San Pablo community, the needs and challenges they faced, community members’ involvement in tourism, and the available resources to support this form of tourism.

**Table 1:** Interview guide to explore key stakeholders’ perspectives on tourism in San Pablo.

No.	Question
**1**	How do you perceive the impact of community-based tourism on the local economic development of San Pablo?
**2**	What is your role or involvement in community-based tourism in San Pablo?
**3**	What are the main objectives or goals you aim to achieve by offering community-based tourism?
**4**	What aspects do you consider most positive or satisfactory in the development of community-based tourism in the community?
**5**	What challenges or difficulties do you face when developing community-based tourism activities in San Pablo?
**6**	What resources (human, financial, infrastructure, etc.) do you currently have to provide community-based tourism services?
**7**	What aspects of community-based tourism do you believe could be improved or added?
**8**	How do community members engage in decision-making and the management of community-based tourism?
**9**	What is the community’s perception of the social and environmental impact of community-based tourism in San Pablo?
**10**	Are there initiatives or programs to promote active community participation in the development and management of community-based tourism?
**11**	How is the promotion and marketing of community-based tourism carried out in San Pablo? What strategies are used to attract visitors and tourists?
**12**	What do you consider the main obstacles or barriers that the San Pablo community faces in the successful implementation of community-based tourism?
**13**	What are the experiences or good practices from other communities in community-based tourism development that you believe could be useful for San Pablo?

Based on the collected data, empathy maps were utilized to gain a deeper understanding of the environment, behavior, aspirations, and concerns of the key users [19]. These data underwent a rigorous analysis process, which encompassed transcription and coding of the interviews, as well as the identification of emerging patterns and themes.

**Fig 1:**
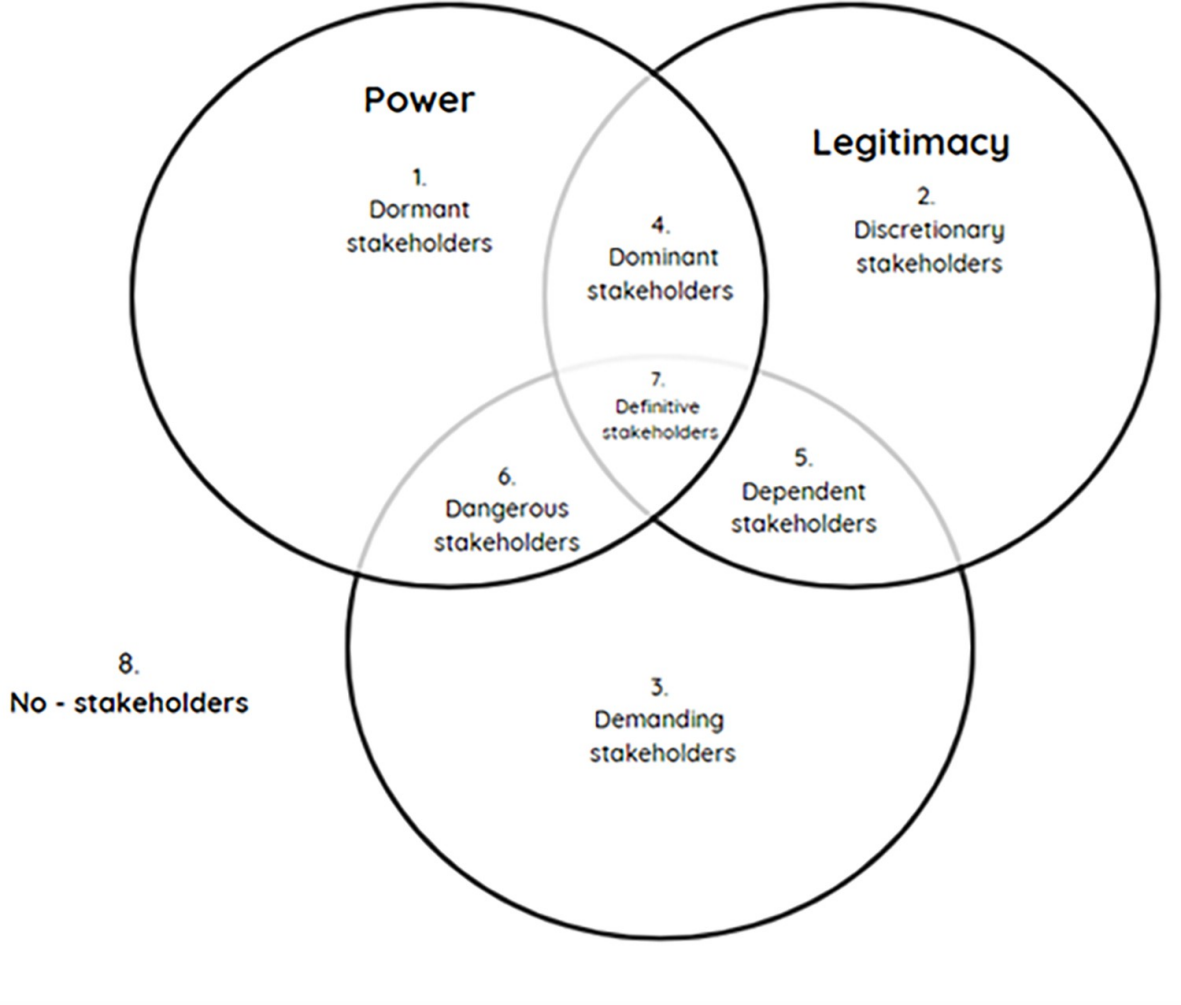
Attributes of stakeholders according to Mitchell et al. [20].

The next tool employed was the Stakeholder Matrix by Mitchell et al. [20], which not only identifies the actors involved in the development of tourism activities in San Pablo but also delineates how these actors can be influenced based on their attributes of power, legitimacy, and urgency concerning the current situation or organization. Building upon these attributes, various stakeholder groups were identified (Fig 1):

- Latent stakeholders, those who have one of the three attributes: dormant, demanding, and discretionary.
- Expectant stakeholders, those who possess two of the attributes: dangerous, dominant, and dependent.
- Definitive stakeholders, who possess all the attributes.

Drawing from this theory, Martins, H. F., & Fontes Filho, J. R. [21] developed a tool to prioritize stakeholder management and allocate appropriate resources to meet their needs and interests. This tool is the Consolidation Matrix (see Table 2), which comprises three matrices:

- Power matrix, which refers to an actor’s ability to impose their will using coercive, utilitarian, and symbolic resources.
- Legitimacy matrix, which indicates whether an actor’s participation is desirable or appropriate for the organization and society.
- Urgency matrix, which refers to the need for immediate attention based on degrees of temporal sensitivity and criticality.

**Table 2:** Consolidation matrix by Martins, H.F. & Fontes, J.R. [21].

CONSOLIDATION MATRIX							
Territory/Location:							
Elaborated by:							
Actors	Level of Power (A)		Level of Legitimacy (B)		Level of Urgency (C)		Total A x B x C
	Total Value	Normalized Value	Total Value	Normalized Value	Total Value	Normalized Value	
Actor 1:							** **
Actor 2:							** **
Actor n:							** **
**TOTAL**							
**Number of actors**							
**Average**							
**Maximum Value**							
**Minimum Value**							
**Central Point**							

The rating used in these matrices is on a scale of 0 to 3, and the total degree of each matrix is obtained by summing the rating values.

#### 3.2.2. Second phase: Analysis and definition

In the subsequent stage of Design Thinking, termed "Analysis and Definition," the information gathered in the previous phase was leveraged to interpret and more precisely define the issues impeding tourism development in the San Pablo community. To achieve this objective, an exhaustive fieldwork was conducted, visiting different tourist attractions in San Pablo, including the main tourist attraction, the natural pool "La Jungla," waterfalls amidst the community’s vegetation, and other relevant sites for community-based tourism. Direct contact was established with key stakeholders in their workplaces and during community meetings to obtain firsthand information.

This direct interaction with community members enabled us to acquire new valuable information, enriching the analysis and providing a deeper understanding of the needs and challenges that San Pablo faces in its endeavor to promote community-based tourism. Furthermore, during the Analysis and Definition phase, the SWOT matrix was applied to analyze both the internal and external situation of the organizational management of tourism activities in San Pablo. This tool proved pivotal in identifying the strengths, weaknesses, opportunities, and threats affecting tourism development in the community, subsequently establishing a robust foundation for decision-making and the formulation of effective solutions.

#### 3.2.3. Third phase: Ideation and prototyping

In the third phase of the Design Thinking process, "Ideation and Prototyping," the focus was on fulfilling objective 2: "Determine an organizational model towards the integration, interrelation, and coordination among stakeholders engaged in community tourism activities." This stage is directly linked to the preceding "Discover and Empathize" and "Analyze and Define" phases.

During the "Ideation and Prototyping" stage, the emphasis was on exploring proposals that addressed the specific needs of the San Pablo community, considering prior knowledge about beneficiaries’ desires and the identification of various involved stakeholders. Furthermore, during this stage, we conducted an additional round of three interviews conducted during a second fieldwork phase. These interviews, conducted by the authors and previously validated by experts, provided us with an in-depth insight into beneficiaries’ perspectives and their ideas for creating effective solutions. During these interviews, we formulated seven key questions (Table 3) for the three beneficiary representatives.

**Table 3:** Interview guide for co-creating solutions with beneficiaries.

No.	Question
**1**	What aspects do you believe could enhance collaboration among individuals and organizations involved in community tourism?
**2**	What resources or support do you think would be useful to strengthen tourism development in the San Pablo community?
**3**	What are the main difficulties you face when participating in community-based tourism activities?
**4**	What ideas come to mind for improving coordination and communication among the stakeholders involved in San Pablo’s tourism?
**5**	What activities or events would you like to see organized to promote interaction and networking among different members of the tourism community?
**6**	What strategies do you think could facilitate the active participation of all community members in the management and development of tourism?
**7**	What ideas or successful practices from other communities do you believe could be adapted and benefit tourism in your community?

These fundamental questions played a crucial role in generating innovative ideas, thereby laying the foundation for prototype design.

#### 3.2.4. Fourth phase: Experimentation and validation

For experimentation and validation, we adopted a participatory approach to gather valuable feedback from the direct users of the project. Initially, we conducted online interviews via the Zoom platform with ten key members of the community involved in tourism activities, including the representative from the local government’s Tourism department in El Triunfo.

During these interviews, we introduced the first low-fidelity prototype, which encompassed the organizational design structure and its components in a general perspective. Within this context, we posed crucial questions to the participants with the aim of capturing their perceptions and contributions (Table 4):

**Table 4:** Interview guide for prototyping feedback and validation with beneficiaries.

No.	Question
**1**	What aspects of the solution do you find most appealing or interesting?
**2**	What aspects do you think could be improved or adjusted to better meet your needs?
**3**	How do you feel when interacting with the solution? Is it intuitive and easy to use?
**4**	What elements of the solution raise doubts or concerns for you?
**5**	Do you believe the proposed solution effectively addresses the identified issues in the community?
**6**	What suggestions or recommendations do you have to enhance integration and coordination among stakeholders involved in community-based tourism?

Based on the insights and suggestions gathered from online interviews, we made improvements to the low-resolution prototype. This led us to embark on a second phase of experimentation and validation, characterized by a more detailed and specific report that comprehensively addressed aspects of the organizational design. In this context, we organized a focus group in the San Pablo community during a field visit. It’s important to note that the participants in the focus group were the same individuals who had previously collaborated in the online interviews.

During this session, we presented the report in both physical and digital formats, giving shape to a high-resolution prototype. Through interactive and dialogic dynamics, we created an environment conducive to in-depth discussion, with the aim of subjecting the proposed solution to comprehensive evaluation. Participants had the opportunity to directly interact with the prototype, immersed in an atmosphere resembling a meeting of the new organization. Furthermore, they could share their perspectives and observations in real-time, significantly enriching the feedback process.

In conjunction with the group sessions, we employed brief surveys to capture quantitative data concerning participants’ perception and satisfaction regarding the final solution. These surveys were validated by five experts in tourism management and communication theories, who confirmed the ethical integrity of the collected data due to its relevance and utility in problem-solving.

## 4. Results

In this section, we present the results of the research employing the Design Thinking methodology for the development of community-based tourism in the San Pablo community, Ecuador. Throughout the process, various stages were undertaken, each yielding important findings that guided our approach towards creating effective and sustainable solutions.

Each of these results, derived from different stages of the aforementioned process, significantly contributed to the creation of the organizational design for the San Pablo community. While this design was specifically tailored to local needs, we emphasize that its general aspects can serve as an example for other community-based tourism initiatives in Ecuador and beyond. These findings represent a step forward towards the sustainable and successful development of community-based tourism across the region. Furthermore, the insights offer valuable contributions to the strategic management of tourism and related enterprises, providing a robust and guiding framework for future initiatives benefiting communities and stakeholders involved in the tourism sector.

### 4.1. Findings from phase one: Discovery and empathy

#### 4.1.1. Needs and/or concerns based on empathy maps

In this section, we present the results obtained from three empathy maps, each corresponding to a representative: one from the Tourism Department of the local government of El Triunfo, one responsible for tourism management in the San Pablo community, and the host of "La Jungla," the main tourist attraction in San Pablo. All three are considered representatives of San Pablo from different roles within the community. We can see the results of the Empathy Maps in the following Table:

The results from Table 5 provided valuable insights that underscore the importance of considering the perspectives and concerns of local stakeholders in the planning and management of tourism in rural communities. The findings highlight the need to establish close collaboration among the various actors involved in tourism development, including community members, governmental and private organizations, as well as visitors. This collaboration has been identified as a critical factor in achieving sustainable and equitable management of tourism resources within the community.

**Table 5:** Key stakeholders’ needs and/or concerns.

Stakeholders	Needs and/or concerns
Local government representative of El Triunfo	Needs to address the lack of coordination and communication within the community to ensure that visitors can enjoy a more successful and mutually beneficial tourism experience.
Tourism management representative of San Pablo	The lack of information and knowledge among locals about providing quality service to tourists is negatively impacting the community’s tourism. Addressing this situation is crucial for improving the visitor experience and the local tourism industry’s development.
Representative of “La Jungla”; one of San Pablo’s main attractions	It’s essential to strike a balance between individually managing tourism services and collaborating with the community to create a comprehensive and appealing tourism product that properly represents both San Pablo and El Triunfo.

#### 4.1.2. Identification and classification of stakeholders

The stakeholders involved in the tourism development of San Pablo were identified as the Local Government of El Triunfo, Cañar, and key individuals within San Pablo’s tourism offerings. With this identification, an analysis of attributes was conducted using the consolidation matrix (see Table 6), based on their participation and cooperation in the community’s tourism activities.

**Table 6:** Consolidation matrix results.

CONSOLIDATION MATRIX							
Territory/Location:	San Pablo Community of El Triunfo						
Elaborated by:	Consulting team						
Actors	Level of power (A)		Level of Legitimacy (B)		Level of Urgency (C)		Total = A x B x C
	Total Value	Normalized Value	Total Value	Normalized Value	Total Value	Normalized Value	
Actor 1: El Triunfo local government (Roxanna Mindiolaza; official from the Tourism department)	14	1,33	5	1,04	6	1,50	**2,08**
Actor 2: Cañar local government	11	1,05	3	0,63	5	1,25	**0,82**
Actor 3: San Pablo community organization (Ángel Rivera; vice president)	11	1,05	4	0,83	3	0,75	**0,65**
Actor 4: Tourism management leader in San Pablo (José Carpio)	13	1,24	6	1,25	4	1,00	**1,55**
Actor 5: Families in charge of animal farming within the community	10	0,95	5	1,04	6	1,50	**1,49**
Actor 6: Owner of “La Jungla” (María Lema)	11	1,05	6	1,25	3	0,75	**0,98**
Actor 7: PWD Committee (Potable Water Board; Moisés Carpio: president)	11	1,05	3	0,63	2	0,50	**0,33**
Actor 8: Transport service provider in the community (Rodrigo)	6	0,57	5	1,04	5	1,25	**0,74**
Actor 9: Responsible for the cave resource (Román Piña)	11	1,05	5	1,04	4	1,00	**1,09**
Actor 10: Academia	7	0,67	6	1,25	2	0,50	**0,42**
**TOTAL**	105		48		40		
**Number of actors**	10		10		10		
**Average**	10,5		4,8		4		
**Maximum Value**	1,33		1,25		1,50		
**Minimum Value**	0,57		0,63		0,50		
**Central Point**	0,95		0,94		1,00		
	2,42						

After completing the consolidation matrix, the range calculations were carried out to categorize the actors, which are presented in Table 7 for better visualization.

**Table 7:** Delimiting ranges for stakeholder classification.

**Power Level**	**LIM—LOW**	**—**	**LIM–UPP**
Lower range	0,57	<x<	0,85
Medium range	0,86	<x<	1,04
Upper range	1,05	<x<	1,33
**Legitimacy Level**	**LIM—LOW **	**— **	**LIM–UPP**
Lower range	0,63	<x<	0,84
Medium range	0,85	<x<	1,03
Upper range	1,04	<x<	1,25
**Urgency Level**	**LIM–LOW **	**— **	**LIM–UPP**
Lower range	0,50	<x<	0,90
Medium range	0,91	<x<	1,09
Upper range	1,10	<x<	1,50

Once the range was established to classify the different types of involved actors, the obtained results were as follows:

- Latent stakeholders, those who have one of the three attributes: dormant, demanding, and discretionary.
- Expectant stakeholders, those who possess two of the attributes: dangerous, dominant, and dependent.
- Definitive stakeholders, who possess all the attributes.

Thus, the final outcome of this mentioned matrix applied to the case of San Pablo is as follows (Table 8)

**Table 8:** Summary of stakeholders involvement matrix.

Actors	Power Level	Legitimacy Level	Urgency Level	Stakeholder Classification
Actor 1: El Triunfo local government (Roxanna Mindiolaza; official from the Tourism department)	UPP	UPP	UPP	DEFINITIVE
Actor 2: Cañar local government	UPP	LOW	UPP	DANGEROUS
Actor 3: San Pablo community organization (Ángel Rivera; vice president)	UPP	LOW	LOW	DORMANT
Actor 4: Tourism management leader in San Pablo (José Carpio)	UPP	UPP	MED	DOMINANT
Actor 5: Families in charge of animal farming within the community	MED	UPP	UPP	DEPENDENT
Actor 6: Owner of “La Jungla” (María Lema)	UPP	UPP	LOW	DOMINANTE
Actor 7: PWD Committee (Potable Water Board; Moisés Carpio: president)	SUP	INF	INF	DORMANT
Actor 8: Transport service provider in the community (Rodrigo)	INF	SUP	SUP	DEPENDENT
Actor 9: Responsible for the cave resource (Román Piña)	SUP	SUP	MED	DOMINANT
Actor 10: Academia	INF	SUP	INF	DISCRETIONARY

**Fig 2:**
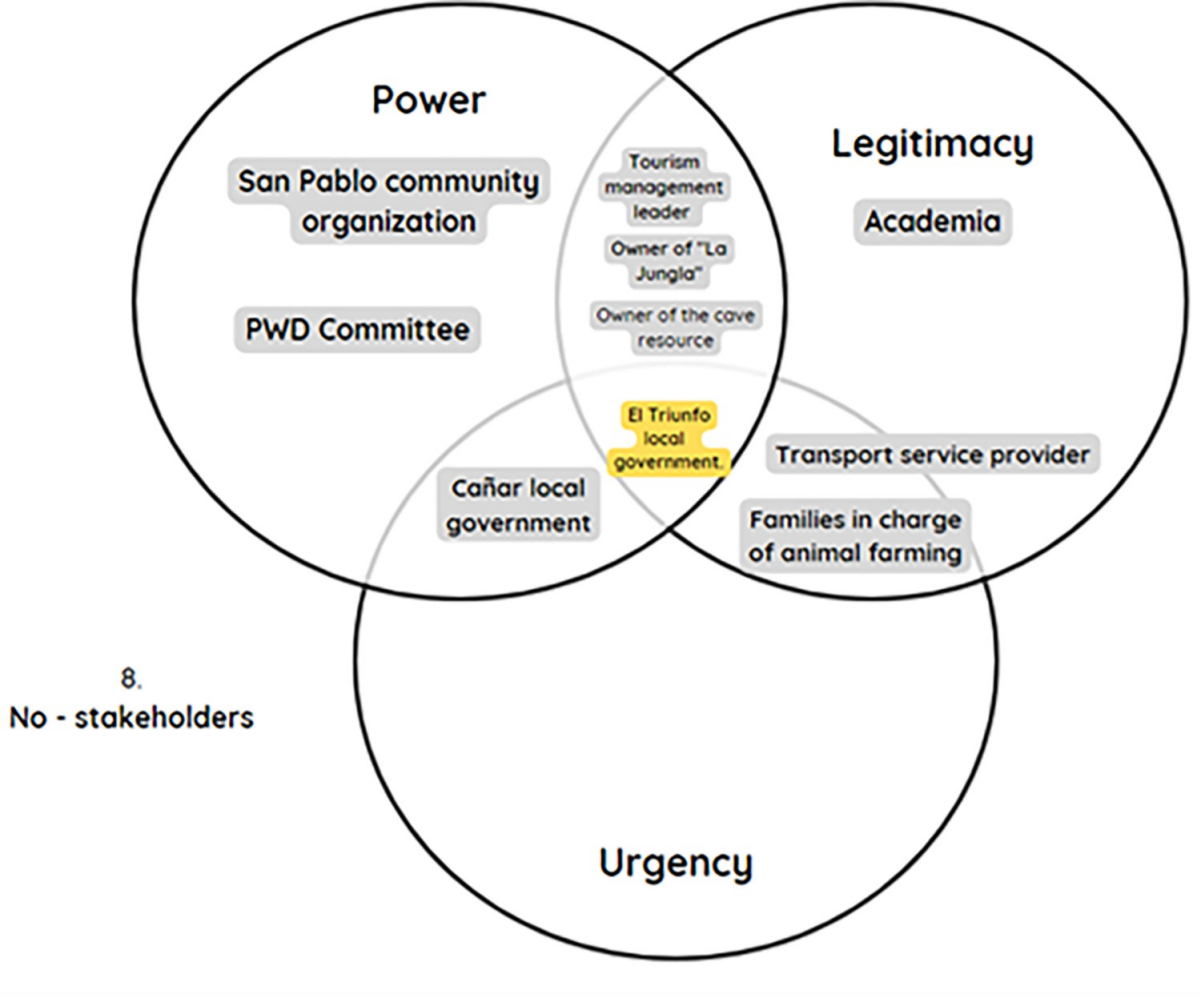
Relationship of actors with attributes.

In detail, as shown in Fig 2, there are different groups of stakeholders involved in San Pablo’s tourism, indicating that each actor has a distinct impact on tourism development. This suggests the need to take special measures to protect their rights and interests and ensure their participation in the process of organizational design, thereby guaranteeing an inclusive, equitable, and sustainable approach.

#### 4.1.3. Description of stakeholder classification in the stakeholder involvement matrix

After analyzing the gathered information and evaluating the degree of influence and power of each actor involved in the present study, each actor has been assigned to a specific category based on their level of impact and capacity to affect the project’s development and outcomes.

The following are the resulting categories in which the stakeholders were classified:

Through this section, the importance of classifying stakeholders in the stakeholders’ matrix is emphasized, as it provides a clear and well-structured perspective of the different actors and their roles in the tourism sector. This information (Table 9) is essential for the formulation of successful management and communication strategies, ensuring effective collaboration and well-informed decision-making that significantly contribute to the success of the research.

**Table 9:** Stakeholders’ roles.

Actor	Type of stakeholders	Role in tourism
El Triunfo local government	Definitive	Possessing all three attributes, their participation is crucial for the success of tourism in the community. It is vital to pay attention to their interests to maximize their engagement and collaboration.
Cañar local government	Dangerous	Due to their power and urgency, it is important to carefully consider their demands and find ways to collaborate with them to achieve common goals.
Responsible for the tourist management of San Pablo, Owner of “La Jungla” and the responsible for the caves resource	Dominant	Their interests influence tourist activity, it is important to establish constant and transparent dialogue with them to ensure their satisfaction and continued engagement.
Transport service provider and families responsible for guinea pig farming	Dependent	Although they do not have decision-making power, their participation is important for the success of tourism. It is necessary to provide them with resources and support to guarantee their commitment and satisfaction.
Community organization and PWB committee	Dormant	Although they have power in the community, their interest in tourism is limited. It is necessary to motivate and engage them in the development of tourism.
Academia	Discretionary	Their participation can be considered at certain moments or in certain decisions. It’s important to maintain open communication and establish strategic alliances with them to leverage their knowledge and experience.

### 4.2. Findings of the second phase: Analysis and definition of criteria

#### 4.2.1. SWOT analysis

To aid in a comprehensive understanding of the SWOT analysis, Table 10 is presented herein.

**Table 10:** SWOT analysis results.

***STRENGTHS***	***WEAKNESSES***
The conducted SWOT analysis has provided a comprehensive view of the situation of the tourism entrepreneurship in San Pablo. An important strength has been identified in the commitment of the tourism entrepreneurs towards the recovery of tourism, as they recognize the value that local natural resources hold for visitors, generating significant opportunities for the benefit of the entire community.	However, weaknesses requiring attention have also been identified. The lack of comprehensive tourism planning is a relevant concern, as it could limit the growth and sustainability of tourism in San Pablo. Likewise, insufficient information and communication are negatively impacting the progress of the local tourism sector, underscoring the need to improve communication channels and access to relevant information for all involved stakeholders.
***OPPORTUNITIES***	***THREATS***
Regarding opportunities, potential strategic alliances with Decentralized Autonomous Governments (GADs), academia, and other neighboring communities stand out. Collaborative efforts and the consolidation of these alliances could enhance the tourism development in the region, fostering the exchange of knowledge, resources, and experiences.	On the other hand, the SWOT analysis has also identified certain threats that must be considered in tourism planning. The irregular territorial limitation of the commune could complicate the equitable management and distribution of tourism resources, necessitating a careful strategy to address this situation.
	Additionally, adverse weather conditions pose a challenge for the development of year-round tourist activities. Exploring alternatives to diversify the tourism offerings and ensuring an appealing experience for visitors even during less favorable periods is important.

### 4.3. Findings of the third phase: Ideation and prototyping

In the "Ideation and Prototyping" phase, concrete ideas were generated based on the responses of the beneficiary representatives and the benchmarking analysis. The focus was on key aspects for organizational design, effective communication channels, strategies to promote collaboration and networking with key stakeholders, resources and support needed to implement and maintain the organizational design, and the measurement and evaluation of its effectiveness in community tourism in San Pablo.

#### 4.3.1. Brainstorming of solution ideas from the perspective of the beneficiaries

In this section, the importance of constructing an appropriate design for community tourism in San Pablo is highlighted, considering the vision and needs of the beneficiaries themselves (Table 11). By emphasizing these aspects, the significance of a participatory and community-centered methodology is underscored to drive the sustainable development of tourism in the region.

**Table 11:** Beneficiaries’ perspectives for building an organizational design.

No.	Question	Beneficiary responses
**1**	What aspects do you think could enhance collaboryation among individuals and organizations participating in community tourism?	• Promoting collaboration among stakeholders. • Establishing alliances with GAD’s, academia, and nearby communities. • Training needs
**2**	What resources or support do you consider would be useful to strengthen the development of tourism in the San Pablo community?	• Training needs. • Improving tourism infrastructure and implementing promotional strategies.
**3**	What are the main challenges you face when participating in community tourism activities?	• Seeking sources of funding. • Facilitating access to knowledge and markets.
**4**	What ideas do you have to improve coordination and communication among the stakeholders involved in tourism in San Pablo?	• Creating a digital communication platform. • Organizing regular meetings. • Forming work groups.
**5**	What activities or events would you like to see held to promote interaction and networking among different members of the tourism community?	• Organizing tourism fairs. • Conducting collaborative workshops. • Designing thematic routes.
**6**	What strategies do you believe could facilitate active participate of all community members in tourism management and development?	• Encouraging inclusion in decision-making. • Providing training opportunities. • Recognizing and valuing contributions.
**7**	What successful ideas or practices from other communities do you think could be adapted and benefit tourism in your community?	• Preserving the autonomy of the community’s natural resources.

#### 4.3.2. Key aspects for the organizational design of San Pablo

Considering each beneficiary perspective and the findings detailed in previous sections, it is essential to design an organizational model that involves various stakeholders in the tourism development of San Pablo while considering their interests and needs. This ensures sustainable development and active, collaborative participation of all involved actors. Therefore, it can be inferred that the organizational design should embrace self-organization as its main pillar. This self-organizational model will allow the creation of a collaboration network where all actors can work together to achieve common objectives. Furthermore, being self-organizational allows different actors to have varying levels of participation and collaboration based on their interests, capabilities, and available resources. This promotes inclusivity and prevents the exclusion of certain stakeholders. Another aspect is its natural adaptability and evolution, providing greater responsiveness to environmental changes and enhanced innovation capacity.

For better understanding, a detailed breakdown of constructs is presented in the following Table 12. These constructs align with the prototypes and serve as a comprehensive guide to develop the appropriate organizational design model, allowing San Pablo’s stakeholders to fully grasp the components of the proposed design.

**Table 12:** Key aspects and constructs for the organizational design of San Pablo.

No.	Key Aspects	Key Constructs
**1**	Inclusion and participation in the organizational structure for the tourism community.	Horizontal organizational structure reflecting real participation and equity between autonomy and responsibility of participants in the community’s tourism activity.
**2**	Strengthening organizational culture and team cohesion.	Self-organizational culture to strengthen the shared identity and values of the tourism community.
**3**	Effective communication channels to interact with stakeholders involved in tourism.	• Establish an open and transparent communication system facilitating information flow among stakeholders. • Utilize technological tools and social networks for effective communication.
**4**	Strategies to promote collaboration and networking with key actors in the regional tourism.	• Create a collaboration network enabling sharing of experiences and resources with nearby tourism communities. • Forge strategic alliances with Decentralized Autonomous Governments and private actors in the tourism sector (Collaborative network work).
**5**	Resources and support needed to implement and maintain the proposed organizational design.	• Establish the organizational structure. • Identify sources of funding and technical support to implement the organizational design. • Reporting strategies.
**6**	Measurement and evaluation of the organizational design’s effectiveness in community tourism in San Pablo.	• Develop performance indicators and assessment metrics to measure the impact of the organizational design.

#### 4.3.3. Horizontal structure organizational chart

**Fig 3:**
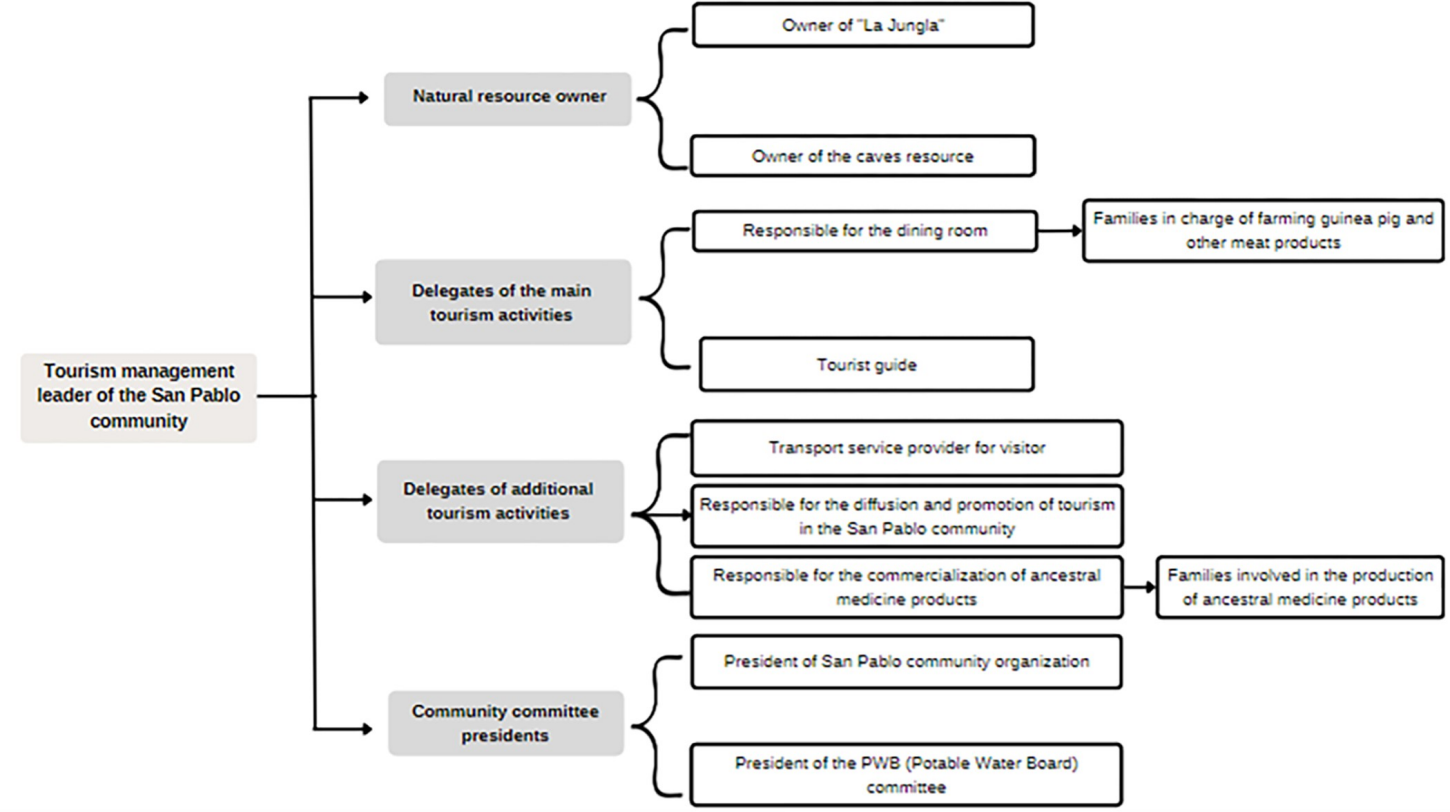
Horizontal organizational chart.

The organizational chart presented in Fig 3 has been designed to strengthen tourism in the San Pablo community. The inclusion of a leader and 8 community members, including natural resource owners, delegates for primary and additional tourism activities, and representatives from community committees, allows all stakeholders to be represented and collaborate effectively in the development of community tourism. It’s worth noting that the inclusion of delegates for tourism activities, such as the responsible person for the dining area and another responsible for the marketing of ancestral medicine products, was added to contribute to a more comprehensive and diversified tourism offering in the community. With this organizational structure, a collaborative and coordinated environment is created where each member has a specific role and responsibility, potentially enhancing the quality and sustainability of tourism. Moreover, they have a voice and a vote in the management and benefits of tourism in their area, which can foster their commitment and empowerment.

#### 4.3.4. Self-organization of the members of the new organizational structure

In a self-organizational structure, roles and functions are less rigid and adapt to the needs of the community. However, it’s still essential to outline responsibilities. Therefore, in Table 13, the specific roles of each member as seen in the organizational chart are presented.

**Table 13:** Responsibilities of stakeholders.

Members	Role, function and/or responsibility
Leader of Tourism Management for the San Pablo Community	Responsible for coordinating the group’s activities and decisions, but their leadership is based on collaboration and consensus with other members rather than imposing authority. Their main function is to encourage teamwork and effective communication among group members.
Natural Resource Owners	Responsible for the care and maintenance of the community’s natural resources. Their role in self-organization is to ensure that visitors respect and care for these resources and collaborate with other members to design tourism activities that promote sustainability and conservation.
Delegates for Primary Tourism Activities	Ensure that activities are carried out effectively and satisfactorily for visitors and collaborate with other members to improve and diversify the tourism offering.
Delegates for Additional Tourism Activities	Contribute to the design of more comprehensive and appealing tourism packages and collaborate with other members to promote and publicize the community’s tourism offering.
Community Committee Presidents	Ensure that tourism decisions and activities align with the interests and needs of the community as a whole. Additionally, they can contribute ideas and proposals to enhance member participation and collaboration in the community’s tourism development.

#### 4.3.5. Media and communication channels

In the context of San Pablo, a community with limited internet access and resources, it’s of vital importance to establish effective and accessible internal communication channels to ensure proper coordination and participation of members in the new organizational structure. The proposed media and communication channels are presented below in the Table 14.

**Table 14:** Media and communication channels to be used in San Pablo.

Media and Communication Channels	Detail
Regular In-Person Meetings	These face-to-face meetings represent a fundamental form of internal communication. They allow direct interaction among members, facilitating collective decision-making and immediate problem-solving. Moreover, these meetings strengthen the sense of community and promote active participation of all involved.
Bulletin Boards and Informational Displays	Placing bulletin boards and informational displays in strategic locations within the community about activities, events, and decisions related to community tourism.
Instant Messaging	Instant messaging applications, such as WhatsApp, are a valuable option to maintain smooth and continuous communication among organization members. Instant messaging allows information sharing, task coordination, and quick and efficient query resolution.

#### 4.3.6. Creation of strategic alliances

With an organizational structure in place, the San Pablo community can:

➢ *Identify synergies and collaboration opportunities*. Having a clear vision of the stakeholders involved in the community’s tourism development and their respective roles and responsibilities, the San Pablo community can identify opportunities to collaborate and generate synergies among different actors.
➢ *Develop a network of contacts*. The community could establish alliances with nearby tourism communities, tourism businesses, or government agencies for the development of joint projects and programs.

Below is Table 15, illustrating how the San Pablo community can benefit once it establishes a strong collaboration network. As mentioned earlier, these partnerships will not only strengthen the community but also contribute to the formulation of policies and strategies for businesses interested in Ecuadorian community tourism.

**Table 15:** Networking efforts of the San Pablo community.

Alliance	Detail	Benefits
Academia	This collaboration could involve knowledge and experience exchange in sustainable tourism project management, as well as conducting joint research to enhance the community’s tourism offerings.	• Establish dialogue and debate spaces with professionals or future professionals. • Contribute to strengthening tourism activities with realistic and innovative proposals, new products, case studies, and especially new experiences.
Alliances with other communities	Partnerships with nearby communities offering community tourism can allow both communities to share resources and knowledge, as well as offer tourism packages to attract more visitors.	• Promote the El Triunfo and its entrepreneurial communities as a preferred coastal destination. • Motivate the development of entrepreneurs’ skills. • Opportunity for job creation.
Alliances with Government Organizations	They can provide access to resources and funding, as well as technical and policy support for the development of community tourism in the region.	This synergy could include actions like improving infrastructure, providing training programs for entrepreneurs, and participating in local events.
Alliances with Environmental Organizations	The community could collaborate with environmental organizations working on the protection and conservation of the environment and natural resources. These organizations could assist the community in improving its environmental management and offering more sustainable tourism experiences.	Development of strategies for urban and natural space renewal linked to environmental recovery of natural areas and enhancing value in communities and tourism enterprises.

#### 4.3.7. Resources and support needed to implement and maintain the proposed organizational design

➢ *Formalization of the organizational structure*. In Ecuador, community tourism has demonstrated the potential to generate economic income and improve the livelihoods of local communities. However, it has been observed that these communities are often perceived and approached as a homogeneous entity, neglecting the importance of considering their individual voices and cultural diversity (5).

Given this, the importance of establishing a formal organizational structure is recognized. In the case of San Pablo, which has opted for a strategy of self-organized tourism, it may consider forming an Association, Cooperative, or Collaboration, considering the legal process and mandatory requirements for social organizations regulated by the Ministry of Tourism of Ecuador. This will contribute to strengthening social fabric and enable a more comprehensive approach to tourism activities through a robust institutional structure, collaboration among members, and facilitating joint coordination of actions.

➢ *Collective funding*. Funding is a cornerstone for the functioning of any organization, whether it’s an association, cooperative, or collaboration. In the case of San Pablo, where a self-organized approach is adopted, financing refers to the community’s capacity to internally obtain necessary resources, without relying heavily on external funds.

Self-organized financing entails the community’s active engagement in generating income from various local economic sources, including tourism activities. Some options for collective financing for community self-organization could include (see Table 16):

**Table 16:** Self-organized funding sources.

Sources of funding	Description	Examples
Income from Tourist Services	The community can generate income by offering tourist services to visitors.	Guided tours to cultural or natural sites, gastronomic experiences with traditional dishes, among other activities.
Community Financing	Raising funds through the community, through events or fundraising campaigns.	Bingos
Cooperatives and Community Ventures	Encourages the creation of local cooperatives and ventures where members engage in joint economic activities.	Production and marketing of local products, crafts, textiles, among others.
Membership Fees	Community members can contribute regular dues to sustain infrastructure and community projects related to tourism.	Monthly fees of $20 per organization member.
Collective Investment	Establish alliances with other communities, businesses, or local actors to develop joint tourism projects and share income generated from tourism.	Local and/or regional tourist routes, joint marketing, and cultural exchange programs.
Sponsorships	Seek sponsors to support the organization’s projects and events.	Tourism and travel companies, financial and environmental services.
Grants	Seek government grants or nonprofit organization grants to support the association’s development.	Local and regional governments.

#### 4.3.8. Activity report

When implementing an organization, it is essential to incorporate an activity reporting system, as this fosters transparency, accountability, communication, and effective planning within the framework of community tourism. Below, in Table 17, the reporting strategies that can contribute to strengthening the development of community tourism in San Pablo can be observed.

**Table 17:** Reporting strategies.

Report	Details
Quarterly organization activity report	Establishing a quarterly report will allow organization members and the community at large to understand the progress and achievements made during that period. This report should include details about conducted tourism activities, generated income, challenges faced, and future plans.
Attendance report for organization meetings	This report will serve to measure the level of participation and commitment of each member to the community initiative. Additionally, it will facilitate the identification of topics or areas that require more attention and will promote greater accountability in attendance and participation by all.
Continuous evaluation and monitoring	Relevant metrics, such as tourist satisfaction, economic impact on the community, and environmental sustainability, must be defined to measure the success and effectiveness of implemented activities.
Transparent financial reports	Financial reports should clearly reflect the income and expenses generated by tourism activities, as well as investments made in local development projects.
Feedback mechanisms	Establishing feedback mechanisms, such as surveys or consultation spaces, will allow community members to express their opinions, concerns, and suggestions regarding community tourism.

### 4.4. Findings of phase four: Experimentation and validation

During this phase of the process, a meticulous validation test of the organizational design was conducted, encompassing all elements previously identified in the constructs. The main objective was to share this proposal with the members of the San Pablo community and analyze its impact on the development of community tourism in the area.

As detailed in the methodology section of the fourth phase, titled "Experimentation and Validation," the solution was presented in the form of a comprehensive report acting as a manual for the implementation and management of the new organizational structure that encompasses all constructs conceived in the earlier stages of ideation and prototyping. Additionally, a final construct that deserves our attention at this moment was incorporated: the "Measurement and Evaluation of Organizational Design Effectiveness" (Table 18). This final construct underscores the importance of ensuring that an appropriate self-organizational structure fosters a robust development of community tourism in San Pablo.

**Table 18:** Measurement and evaluation tools.

Detail	Performance Indicators
Tourist Influx	Tourist Satisfaction
Community Participation	Local Collaboration
Local Economic Impact	Community Image Regeneration
Model Replicability	Member Satisfaction
Satisfaction Degree	Commitment and Participation
Identification of Challenges	Communication and Transparency
Networks and Partnerships	Number of Agreements Reached
Impact on Tourist Offer	Benefits for Allies
Visibility Improvement	Durability of Alliances

#### 4.4.1. Measurement and evaluation of organizational design effectiveness

It’s important to mention that data collection and analysis will be conducted periodically, allowing for the observation of trends and changes over time. The feedback obtained from surveys, opinions, and perceptions will be essential for adjusting, improvements, and adaptations to the organizational design, thus ensuring appropriate strategies based on the obtained results.

#### 4.4.2. Perception and acceptance of organizational design

Finally, we address the perception and acceptance of the organizational design through five key aspects (Table 19) that encompass everything contributing to the successful implementation of this structure, based on the principles of self-organization and networked collaboration.

**Table 19:** Questions to assess perception and acceptance of the organizational design among community members.

Aspect to Evaluate	Question	Score
Facilitation of collaboration and teamwork among San Pablo stakeholders.	To what extent do you consider that the organizational design would facilitate collaboration and teamwork among the stakeholders involved in community tourism?	5
Determination of responsibilities for each member within the organizational design.	How do you value the determination of responsibilities for each member within the organizational design?	5
Ability of the organizational design to create strategic partnerships with other organizations and entities.	How do you value the ability of the organizational design to create strategic partnerships with other organizations and entities?	4,66
Contribution of the organizational design to the sustainable development of community tourism.	To what extent do you consider that the organizational design would contribute to the sustainable development of community tourism in the community?	4
Effectiveness of communication channels used in the organizational structure for decision-making and activity coordination.	How do you value the effectiveness of the communication channels that would be used within the organizational structure for decision-making and activity coordination?	4,33
Creation of new opportunities	Do you believe that the implementation of the organizational model could lead to the identification of new opportunities for projects involving private actors in the tourism sector?	4,56

These aspects were explored through specific questions directed at the beneficiaries of this study. The resulting assessment provided us with a deeper understanding of how these key elements are perceived and integrated into the organizational design, enriching our understanding of their impact on community tourism, and supporting the successful implementation of this proposal.

In relation to the validation of the organizational design, we conducted a survey consisting of 7 questions targeted at a group of 10 representatives actively involved in tourism activities in San Pablo. We used a 5-point Likert Scale, where 1 represents "Very Poor" and 5 indicates "Very Good." The results obtained from these surveys, administered to key stakeholders in the community’s tourism activities, reveal certain significant patterns.

It’s noteworthy that the highest scores were assigned to aspects related to the organizational design’s ability to foster strategies that promote collaboration and teamwork. Similarly, an outstanding rating was given to the construct related to individual responsibilities of members within the organization. This demonstrates that despite their different roles, all converge toward a common goal: the collective benefit of the community.

A significant, though to a lesser extent, value was also given to the organizational design’s ability to establish partnerships with other stakeholders that would enhance the community’s competitiveness in the national tourism market. Participants greatly appreciated the suitability of the proposed communication channels, deeming them appropriate to meet the specific needs of the organization.

Regarding the aspects that received comparatively lower scores, the contribution to sustainable development stands out. This could be attributed to economic and legal challenges that have not yet been resolved. However, in subsequent discussions, it was emphasized that the presented manual holds considerable value as it guides the community towards effective self-management using available resources.

These results underscore the importance of further strengthening collaboration, promoting understanding of individual responsibilities, fostering strategic alliances, and advancing on the path of sustainable development in tourism activities. The responses provide valuable insights for the continuous improvement of the organizational design and its effective implementation within the context of San Pablo.

## 5. Conclusion

In summary, this study has addressed the critical need to equip communities with effective tools for the development and management of community tourism in Ecuador. Despite the existence of documents and academic sources highlighting the importance of essential strategic components such as self-organization and territorial networks, a significant gap has been identified in practical knowledge about how to implement and manage these concepts in the specific context of communities, as seen in the case of the San Pablo community. While this community possesses valuable natural and cultural resources, it has faced challenges due to the lack of an effective organizational structure to guide its tourism efforts.

This study has presented a series of key aspects that provide a roadmap for the implementation of a self-organizational design, backed by experts and relevant academic sources. Through the creation of a detailed report and the validation of its constructs, a comprehensive strategy has been outlined to strengthen community tourism in San Pablo and, by extension, in other similar communities.

The obtained results have reaffirmed the necessity to consider the perspectives and concerns of local stakeholders in the planning and management of tourism in rural contexts. The inherent value of close collaboration among individuals involved in tourism development has been underscored. The proposed self-organizational model, supported by academic evidence and internal stakeholders’ acceptance in San Pablo, has the potential to enhance the quality of information and coordination among stakeholders, promoting participation and cooperation.

It’s crucial to highlight that the effective management of a horizontal organizational structure relies on careful member selection and the clear definition of their roles and responsibilities. Open and constant communication among selected members, whether in the form of cooperatives, collaboration, or partnership, is a fundamental pillar for ongoing success. Regular performance evaluation is also essential to drive continuous improvements in community tourism management.

It’s necessary to acknowledge the inherent limitations in any study, and this one is no exception. Despite our efforts to comprehensively address the approaches, some perspectives might not have been fully covered. Among these considerations are unforeseen economic changes or fluctuations in sociopolitical conditions that could influence the viability of community tourism initiatives; the involvement of external stakeholders; and, in the case of other communities seeking to replicate this design, the importance of considering their cultural richness and unique context.

In closing, this study has the potential to generate an impact not only on the San Pablo community but also on the management of tourism strategies by companies and other entities involved in community tourism across Ecuador. The effective implementation of the self-organizational design supported by this research can lead to significant improvements in various aspects. Companies operating in the tourism sector within communities can learn valuable lessons about participatory management and collaborative networking. By adopting approaches that foster collaboration among local stakeholders and the community, companies can benefit from increased local support, sustainable resource utilization, and a more authentic and appealing tourist offering for visitors. Involvement in community tourism initiatives can also enhance corporate image by demonstrating commitment to local and sustainable development.

## Supporting information

S1 File (DOCX)

## References

[1] Rodas M, Donoso NU, Sanmartín I. Community Tourism in Ecuador: A literature review. Tourism, Development and Good Living Magazine. 2015;(9):60–78. https://dialnet.unirioja.es/servlet/articulo?codigo=5309454

[2] UNWTO. Tourism Definitions. 2019; https://www.e-unwto.org/doi/book/10.18111/9789284420858

[3] Spenceley A, Meyer D. Tourism and poverty reduction: Theory and practice in less economically developed countries. J Sustain Tour. 2012;20(3):297–317. doi: 10.1080/09669582.2012.668909

[4] Bravo O, Zambrano P. Community tourism from the local development perspective: a challenge for the Comuna 23 de Noviembre, Ecuador. Espacios Magazine. 2018;39(7):28–43. https://revistaespacios.com/a18v39n07/a18v39n07p28.pdf

[5] Ruiz E, Hernández M, Coca A, Cantero P, Del Campo A. Community tourism in Ecuador. Understanding community-based tourism from the community. Pasos Tourism and cultural heritage magazine. 2008;6(3):399–418. doi: 10.25145/j.pasos.2008.06.031

[6] Richards G, Hall D. Tourism and Sustainable Community Development. Vol. 2. London: Routledge; 2003.

[7] Pilving T, Kull T, Suškevics M, Viira AH. The tourism partnership life cycle in Estonia: Striving towards sustainable multisectoral rural tourism collaboration. Tour Manag Perspect. 2019; 31:219–30. 10.1016/j.tmp.2019.05.001

[8] Nielsen BB. The role of trust in collaborative relationships: A multi-dimensional approach. Management. 2004;7(3):239–56. 10.3917/mana.073.0239#xd_co_f=ZmJmNTRkZDYtZjQyNy00ZDhlLThlYjAtYTY2NzIwMzZhNjgx~

[9] Gajdošík T, Gajdošíková Z, Maráková V, Borseková K. Innovations and networking fostering tourist destination development in Slovakia. Quaestiones Geographicae. 2017;36(4). 10.1515/quageo-2017-0039

[10] Kimbu AN, Ngoasong MZ. Centralised decentralisation of tourism development: A network perspective. Ann Tour Res. 2013; 40:235–59. 10.1016/j.annals.2012.09.005

[11] Reyes M., Ortega Á., & Machado E. Model for the integrated management of community tourism in Ecuador, Pastaza case study. REVESCO. 2017;(123):250–75. 10.5209/REVE.53242

[12] Garbanzo-Vargas GM. Organizational Development and Change Processes in Educational Institutions, a Challenge for The Management of Education. Education magazine. 2015;40(01):67–87. 10.15517/revedu.v40i1.22534

[13] Hellriegel D, Jackson SE, Slocum JW. Management, a competency-based approach. 12th ed. Mexico DF: Cengage Learning Editors, SA. 2005.

[14] Leshnower S. Teaching leadership. Gifted Child Today. 2008;31(2):29–35. 10.4219/gct-2008-764

[15] Paül V. Self-organization in tourism: Power to the people. Ann Tour Res. 2010;671–91. 10.1016/j.annals.2010.01.006

[16] Pavlovich K. A rhizomic approach to tourism destination evolution and transformation. Tour Manag. 2014; 41:1–8. 10.1016/j.tourman.2013.08.004

[17] Rodríguez RM, Fernández JIP. Tourism development and relational dynamics. Analysis methodology for the active management of tourist destinations. Tourism notebooks. 2009;(23):173–94. https://revistas.um.es/turismo/article/view/70091

[18] Dam R, Siang T. Interaction Design Fundation. 2022. What is Design Thinking and Why Is It So Popular?. https://www.interaction-design.org/literature/article/what-is-design-thinking-and-why-is-it-so-popular

[19] Ferreira B, Silva W, Oliveira E, Conte T. Designing Personas with Empathy Map. In: SEKE. 2015. https://www.researchgate.net/profile/Bruna-Ferreira-7/publication/276207468_Designing_Personas_with_Empathy_Map/links/5552b16208aeaaff3bf00076/Designing-Personas-with-Empathy-Map.pdf

[20] Mitchell RK, Agle BR, Wood DJ. Toward a theory of stakeholder identification and salience: Defining the principle of who and what really counts. Acad manage rev. 1997;22(4):853–86. 10.5465/amr.1997.9711022105

[21] Martins HF, Fontes Filho JR. On whom is the focus? Identifying stakeholders for the formulation of the organizational mission. CLAD Magazine Reform and Democracy. 1999;15(15):1–18. https://www.academia.edu/download/58562460/Falcao___Fontes._En_quien_se_pone_el_Foco.pdf

