# Supplementary material for: Organizational design for strengthening community-based tourism: Empowering stakeholders for self-organization and networking
Source: PLoS One. 2024 Jan 23;19(1):e0294849. doi: 10.1371/journal.pone.0294849 (PMC10805285; doi:10.1371/journal.pone.0294849)
Supplement: S1 File — (DOCX) [file pone.0294849.s001.docx]

**Organizational Design for Strengthening Community-Based Tourism: Empowering Stakeholders for Self-Organization and Networking**

**Natalia Romero–Medina^1^, Emily Flores–Tipán^1^, Mauricio Carvache-Franco^2^, Orly Carvache-Franco^3^, Wilmer Carvache-Franco^1^*, Ruth González-Núñez^4^**

^1^ Facultad de Ciencias Sociales y Humanísticas, Escuela Superior Politécnica del Litoral, ESPOL, Guayaquil, Ecuador

^2^Universidad Espíritu Santo, Ecuador, Samborondón, Ecuador

^3^Facultad de Economía y Empresa, Universidad Católica de Santiago de Guayaquil, Guayaquil, Ecuador

^4^Facultad de Ciencias Sociales, Educación comercial y Derecho, Universidad Estatal de Milagro, Milagro, Ecuador

Corresponding Author *

**Interview Guide**

**Interviews with the main beneficiaries of the project to gather information about the San Pablo community and how to operate within the tourism sector.**

| **Formal Interview directed to members of the local government of the El Triunfo** |
| --- |
| **TOPIC:** **Organizational Design for Strengthening Community-Based Tourism: Empowering Stakeholders for Self-Organization and Networking**  Date: __/__/____  **Interviewee’s name:**  **Company or Community:**  **OBJECTIVES:**  I) Analyze the organizational situation of the San Pablo community to identify the present organizational and communicational challenges and needs within the tourism sector, and II) Determine an organizational model that promotes self-organization, integration, and coordination among stakeholders engaged in the community's tourism activities.  **QUESTIONS:**  **Local government of El Triunfo and the tourism**   1. Since when has the tourism department been established within the local government of the El Triunfo? 2. What role does Tourism play in the strategic management of the local government? Could you expound on the underlying tourism proposition? 3. How would you characterize the significance of tourism in the city? Do you perceive interconnections with other economic activities, and if so, which ones? 4. Does the Tourism department maintain a comprehensive database, and is it readily accessible? 5. What is your assessment of the local community's disposition towards the advancement of tourism activities? 6. Could you elaborate on the inflow of visitors to El Triunfo? What demographic does this encompass? Furthermore, from where does the primary demand originate, and are there discernible patterns in the timing and nature of tourism consumption? 7. How sizeable is the population engaged in tourism-related activities? Additionally, has the municipality systematized this demographic data, and if so, how may one access it? 8. In comparison to other productive entrepreneurship, what role does tourism play in creating employment opportunities for the canton? 9. Could you elucidate the decision-making process concerning tourism activities within the purview of the local government?   **At an Organizational level**   1. What communication channels are employed by this local government to disseminate information and facilitate engagement? 2. How do you evaluate the current modality of information exchange between the public and private sectors concerning tourism management? 3. In your perspective, is the organization of activities in El Triunfo driven by individual entities or a collective organization involving various territories? 4. Does El Triunfo, as an organization, possess a well-defined structural framework? If so, is there a consensus on the roles assigned within the organization? 5. From your vantage point, what shortcomings have you identified in the transmission of information between distinct productive sectors within the canton?   **About San Pablo Community**   1. Is there an established collaboration between the public sector and tourism entrepreneurs in the San Pablo community? If affirmative, could you delineate the nature of this collaboration and the frequency of communication? 2. Are training programs conducted for tourism entrepreneurs in the canton? If so, could you provide insights into the nature, coordination, duration, and modality of these programs? 3. In your estimation, to what extent is the San Pablo community integrated into the development and organization of tourism activities? 4. Does the San Pablo community actively participate in political decision-making processes related to tourism? If yes, what is the nature of this involvement? |

| **Formal Interview with a Leader of the San Pablo Community Organization** |
| --- |
| **TOPIC:** **Organizational Design for Strengthening Community-Based Tourism: Empowering Stakeholders for Self-Organization and Networking**  Date: __/__/____  **Interviewee’s name:**  **Company or Community:**  **OBJECTIVES:**  I) Analyze the organizational situation of the San Pablo community to identify the present organizational and communicational challenges and needs within the tourism sector, and II) Determine an organizational model that promotes self-organization, integration, and coordination among stakeholders engaged in the community's tourism activities.  **QUESTIONS:**  **Community Organization and the Tourism**   1. Since what year has the current organization been in operation? Until when will it be effective? 2. How is the organization structured? What role does each member play? 3. What position does the community organization hold in relation to tourism? 4. Have they implemented any projects for the tourism development of the San Pablo community? 5. What motivates the pursuit of tourism development in the community? 6. How does the community organization regulate tourism establishments within the community? 7. What is your opinion on the local population's attitude towards the development of tourism activities? 8. Where do the people who visit the canton and the San Pablo community come from? 9. What advantages and difficulties do you encounter in the community? 10. Do you believe that the San Pablo community is integrated into the development and organization of tourism activities?   **Community Organization and other actors in the tourism activity**   1. How would you describe your interaction with the local government of El Triunfo? 2. Does the San Pablo community participate in political decision-making related to tourism? How? 3. Do you recognize other actors participating in tourism activities in the community? Do they have relationships with actors from those sectors? 4. Which sector brings the most economic benefits to the community?   **Community Organization and its Perspective on an Organizational Structure with Self-Organization and Networking Capabilities**   1. Have you heard about "Networking"? 2. What is your opinion on applying "Networking" in the organization of your community? 3. Which individuals or groups, organizations, and/or associations do you believe should be more involved in tourism activities? |

| **Formal Interview directed to an Individual involved in Tourism-related Activities in the San Pablo Community** |
| --- |
| **TOPIC:** **Organizational Design for Strengthening Community-Based Tourism: Empowering Stakeholders for Self-Organization and Networking**  Date: __/__/____  **Interviewee’s name:**  **Company or Community:**  **OBJECTIVES:**  I) Analyze the organizational situation of the San Pablo community to identify the present organizational and communicational challenges and needs within the tourism sector, and II) Determine an organizational model that promotes self-organization, integration, and coordination among stakeholders engaged in the community's tourism activities.  **QUESTIONS:**  **About the Tourism:**   1. From your perspective, does the community benefit from the tourism activities undertaken? 2. Have you received any training from the local government of El Triunfo? 3. Are you aware of any projects that the local government has to promote tourism in the canton? 4. Do you believe it is necessary to make decisions in collaboration with the local government? 5. Does your community have a relationship with the local government El Triunfo? If the answer is positive, how would you describe your community's relationship with the local government? Why? 6. How is your relationship with other stakeholders involved in tourism activities? 7. How is your relationship with other productive sectors in the city?   **Community Organization and its Relationship with Other Actors involved in Tourism Activities**   1. How do you perceive the attitude of the community towards the tourism development of the area? 2. What motivated you to get involved in the tourism growth of the community, and what motivates you to continue in this sector? 3. What challenges do you encounter in community organization? 4. What actions do you believe should be taken for the successful development of tourism in the community? 5. Are you familiar with the current organizational structure of the community? 6. Are you involved in decision-making within the community? 7. Do you receive any financial incentives from the local government and/or private organizations? 8. What are your thoughts on the environmental situation in the community? 9. Do you believe that organized social groups can benefit communities in their long-term existence?   **Community Organization and its Perspective on an Organizational Structure with Self-Organization and Networking Capabilities**   1. Have you heard about "Networking"? 2. What is your opinion on applying "Networking" in the organization of your community? 3. Which individuals or groups, organizations, and/or associations do you believe should be more involved in tourism activities? 4. What are your thoughts on collaborating with other productive sectors in the city? |

| **Interview Guide for Co-creating solutions with Beneficiaries** |
| --- |
| **TOPIC:** **Organizational Design for Strengthening Community-Based Tourism: Empowering Stakeholders for Self-Organization and Networking**  Date: __/__/____  **Interviewee’s name:**  **Company or Community:**  **OBJECTIVES:**  I) Analyze the organizational situation of the San Pablo community to identify the present organizational and communicational challenges and needs within the tourism sector, and II) Determine an organizational model that promotes self-organization, integration, and coordination among stakeholders engaged in the community's tourism activities.  **QUESTIONS:**   1. How do you perceive the impact of community-based tourism on the local economic development of San Pablo? 2. What is your role or involvement in community-based tourism in San Pablo? 3. What are the main objectives or goals you aim to achieve by offering community-based tourism? 4. What aspects do you consider most positive or satisfactory in the development of community-based tourism in the community? 5. What challenges or difficulties do you face when developing community-based tourism activities in San Pablo? 6. What resources (human, financial, infrastructure, etc.) do you currently have to provide community-based tourism services? 7. What aspects of community-based tourism do you believe could be improved or added? 8. How do community members engage in decision-making and the management of community-based tourism? 9. What is the community's perception of the social and environmental impact of community-based tourism in San Pablo? 10. Are there initiatives or programs to promote active community participation in the development and management of community-based tourism? 11. How is the promotion and marketing of community-based tourism carried out in San Pablo? What strategies are used to attract visitors and tourists? 12. What do you consider the main obstacles or barriers that the San Pablo community faces in the successful implementation of community-based tourism? 13. What are the experiences or good practices from other communities in community-based tourism development that you believe could be useful for San Pablo? |

| **Interview Guide for Co-creating solutions with Beneficiaries** |
| --- |
| **TOPIC:** **Organizational Design for Strengthening Community-Based Tourism: Empowering Stakeholders for Self-Organization and Networking**  Date: __/__/____  **Interviewee’s name:**  **Company or Community:**  **OBJECTIVES:**  I) Analyze the organizational situation of the San Pablo community to identify the present organizational and communicational challenges and needs within the tourism sector, and II) Determine an organizational model that promotes self-organization, integration, and coordination among stakeholders engaged in the community's tourism activities.  **QUESTIONS:**   1. What aspects do you believe could enhance collaboration among individuals and organizations involved in community tourism? 2. What resources or support do you think would be useful to strengthen tourism development in the San Pablo community? 3. What are the main difficulties you face when participating in community-based tourism activities? 4. What ideas come to mind for improving coordination and communication among the stakeholders involved in San Pablo's tourism? 5. What activities or events would you like to see organized to promote interaction and networking among different members of the tourism community? 6. What strategies do you think could facilitate the active participation of all community members in the management and development of tourism? 7. What ideas or successful practices from other communities do you believe could be adapted and benefit tourism in your community? |

| **Interview Guide for Prototyping Feedback and Validation with Beneficiaries** |
| --- |
| **TOPIC:** **Organizational Design for Strengthening Community-Based Tourism: Empowering Stakeholders for Self-Organization and Networking**  Date: __/__/____  **Interviewee’s name:**  **Company or Community:**  **OBJECTIVES:**  I) Analyze the organizational situation of the San Pablo community to identify the present organizational and communicational challenges and needs within the tourism sector, and II) Determine an organizational model that promotes self-organization, integration, and coordination among stakeholders engaged in the community's tourism activities.  **QUESTIONS:**   1. What aspects of the solution do you find most appealing or interesting? 2. What aspects do you think could be improved or adjusted to better meet your needs? 3. How do you feel when interacting with the solution? Is it intuitive and easy to use? 4. What elements of the solution raise doubts or concerns for you? 5. Do you believe the proposed solution effectively addresses the identified issues in the community? 6. What suggestions or recommendations do you have to enhance integration and coordination among stakeholders involved in community-based tourism? |

| **Interview Guide for Prototyping Feedback and Validation with Beneficiaries** |
| --- |
| **TOPIC:** **Organizational Design for Strengthening Community-Based Tourism: Empowering Stakeholders for Self-Organization and Networking**  Date: __/__/____  **Interviewee’s name:**  **Company or Community:**  **OBJECTIVES:**  I) Analyze the organizational situation of the San Pablo community to identify the present organizational and communicational challenges and needs within the tourism sector, and II) Determine an organizational model that promotes self-organization, integration, and coordination among stakeholders engaged in the community's tourism activities.  **QUESTIONS:**   1. To what extent do you consider that the organizational design would facilitate collaboration and teamwork among the stakeholders involved in community tourism? 2. How do you value the determination of responsibilities for each member within the organizational design? 3. How do you value the ability of the organizational design to create strategic partnerships with other organizations and entities? 4. To what extent do you consider that the organizational design would contribute to the sustainable development of community tourism in the community? 5. How do you value the effectiveness of the communication channels that would be used within the organizational structure for decision-making and activity coordination? 6. Do you believe that the implementation of the organizational model could lead to the identification of new opportunities for projects involving private actors in the tourism sector? |
